# Bioactive Compounds in *Aegopodium podagraria* Leaf Extracts and Their Effects against Fluoride-Modulated Oxidative Stress in the THP-1 Cell Line

**DOI:** 10.3390/ph14121334

**Published:** 2021-12-20

**Authors:** Karolina Jakubczyk, Agnieszka Łukomska, Sylwester Czaplicki, Anna Wajs-Bonikowska, Izabela Gutowska, Norbert Czapla, Małgorzata Tańska, Katarzyna Janda-Milczarek

**Affiliations:** 1 Department of Human Nutrition and Metabolomics, Pomeranian Medical University in Szczecin, ul. Broniewskiego 24, 71-460 Szczecin, Poland; agnieszka_lukomska@wp.pl (A.Ł.); katarzyna.janda.milczarek@pum.edu.pl (K.J.-M.); 2 Department of Plant Food Chemistry and Processing, Faculty of Food Sciences, University of Warmia and Mazury in Olsztyn, Pl. Cieszyński 1, 10-726 Olsztyn, Poland; sylwester.czaplicki@uwm.edu.pl (S.C.); m.tanska@uwm.edu.pl (M.T.); 3 Institute of Natural Products and Cosmetics, Faculty of Biotechnology and Food Sciences, Lodz University of Technology, Stefanowskiego 2/22, 90-537 Lodz, Poland; anna.wajs@p.lodz.pl; 4 Department of Medical Chemistry, Pomeranian Medical University in Szczecin, ul. Powstancow Wlkp 72, 71-460 Szczecin, Poland; gutowska@pum.edu.pl; 5 Clinic of Plastic, Endocrine and General Surgery, Pomeranian Medical University in Szczecin, 72-009 Police, Poland; norbertczapla@gmail.com

**Keywords:** natural compounds, antioxidant, oxidative stress, goutweed, fluoride, polyphenols

## Abstract

*Aegopodium podagraria* L. (goutweed), a member of the *Apiaceae* family, is a common perennial plant found all around the world that has been used in folk medicine since antiquity. Goutweed leaves contain polyacetylenes, essential oils, mono- and sesquiterpenes, vitamins, macro- and microelements, and phenolic compounds. In spite of its many health-promoting properties, including antioxidant effects, the plant has not been thoroughly studied. The aim of this study was to investigate the antioxidant properties of different goutweed leaf extracts and their effects on the THP-1 cell line, and also to describe the chemical characteristics of goutweed. Falcarinol and falcarindiol and essential oil were determined by gas chromatography coupled with mass spectrometry. Spectrophotometry was used to measure the total content of polyphenols and antioxidant activity–by DPPH and FRAP methods. Oxidative stress in THP-1 cells was induced via sodium fluoride. Then, goutweed leaf extracts were added to evaluate their influence on antioxidant potential (ABTS) and the activity of antioxidant enzymes. Confocal microscopy was used to visualise the production of cytoplasmic and mitochondrial reactive oxygen species (ROS) and for in vitro imaging of apoptosis. The ethanol extracts have a high total content of polyphenols, polyacetylenes, and essential oil, as well as high antioxidant potential. The main volatiles represented diverse chemical groups, which are both oxygenated derivatives of sesquiterpenes and monoterpenes. We also demonstrated positive effects of the high antioxidant potential and increased activity of antioxidant enzymes on cell cultures under severe fluoride-induced oxidative stress. Extraction at 80 ℃ and the use of ethanol as a solvent increased the antioxidant capacity of the extract. The leaves of *Aegopodium podagraria* may serve as a valuable source of antioxidants in the daily diet and assist in the prevention and treatment of oxidative stress-mediated conditions, e.g., inflammatory conditions, cardiovascular diseases, neurodegenerative diseases, and even obesity.

## 1. Introduction

**Figure 1:**
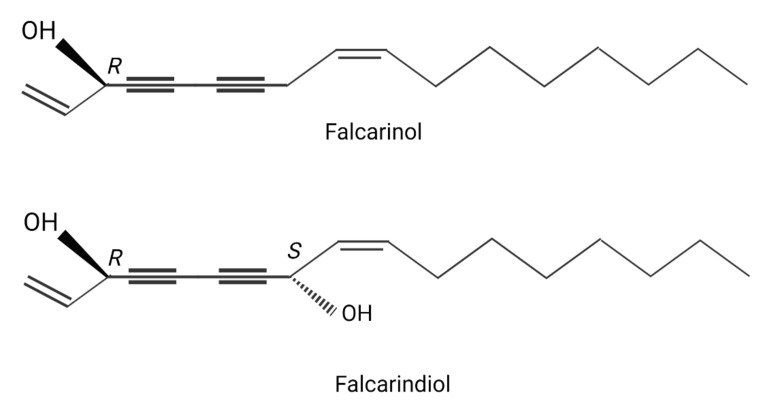
Chemical structures of the polyacetylenes falcarinol [(3*R*)-heptadeca-1,9(*Z*)-diene-4,6-diyne-3-ol] and falcarindiol [(3*R*,8S)-heptadeca-1,9(*Z*)-diene-4,6-diyne-3,8-diol]. Created with BioRender.com.

Common goutweed (*Aegopodium podagraria* L.), a member of the *Apiaceae* family, is a common perennial plant found all around the world, including Europe, North America, and Asia [1,2]. It spreads readily via creeping rhizomes and stolons, as well as being frost hardy. Goutweed is also highly competitive against other species, rapidly taking over the land, which makes it easy to source. Goutweed leaves have been used in folk medicine since antiquity. Infusions made from dried leaves are recommended to remedy gout, haemorrhoids, inflammatory conditions of the kidneys and bladder, as an auxiliary treatment for kidney stone disease, and to improve metabolism. Fresh leaves can be put on wounds to facilitate healing [1,2,3]. Apart from its medicinal properties, goutweed is also an edible plant. New leaves were traditionally eaten in the spring and put into soups, often together with other plants. In many countries, leaves are used in the form of infusions, extracts, and supplements. Goutweed leaves contain polyacetylenes, including falcarinol and falcarindiol, essential oils, chiefly mono- and sesquiterpenes, vitamins, as well as macro- and microelements, notably iron, copper, manganese, titanium, boron, calcium and potassium [3,4,5]. Phenolic compounds, including coumarins, phenolic acids, and flavonoids, can also be found in goutweed [1,2,3,4,5]. Falcarinol and falcarindiol are the main biologically active compounds naturally occurring in plants of the *Apiaceae* family, both wild, e.g., goutweed, and in vegetables such as carrots, parsley, and celery. The amount of these compounds depends on many factors, including the species and part of the plant. They belong to the class of organic compounds known as long-chain *fatty alcohols*. Falcarinol is a polyyne with two carbon–carbon triple bonds and two double bonds (Figure 1). By influencing prostaglandin metabolism, falcarinol has anti-inflammatory and anticoagulant properties. Its concentration-dependent antioxidant properties have also been demonstrated [6,7].

According to the available literature, *Aegopodium podagraria* is safe to use [1,2,3]. This is because of the wide margin between the therapeutic and toxic dose of the main active compound in the plant (falcarinol), which makes it possible to use the product without the risk of adverse effects [4]. On the other hand, there is a shortage of scientific reports confirming the safety of extracts in vitro and in vivo, as well as detailed biochemical analyses of goutweed preparations and their health benefits.

That is why the aim of this study was to investigate the antioxidant properties of different goutweed leaf extracts and their effects on the THP-1 cell line and also to describe goutweed characteristics in terms of the content of essential oils, total polyphenols, and polyacetylenes—the main active compounds found in the plant. Additionally, we examined the effects of solvent DMSO (dimethylsulfoxide) at a concentration of 1% the volume of the cell medium, comparing it to standard solvents, that is water and ethanol, and studied the effects of extracts against sodium fluoride toxicity in the THP-1 cell line. The monocytes of the THP-1 cell line easily differentiate into macrophages after treatment with phorbol ester, and the process itself is fast and takes only 24 h. Macrophages are a major source of reactive oxygen species, reactive nitrogen species, and peroxynitrite generated through the so-called respiratory burst. Constitutively released pro-inflammatory cytokine, e.g., tumour necrosis factor-α, triggers nuclear factor-κB and activator protein-1 translocation, leading to the overproduction of reactive oxygen species and reactive nitrogen species in macrophages. Additionally, tissue-resident macrophages present in the liver, lung, skin, kidney, bone, and connective tissue act as non-specific killer cells that eliminate bacteria, foreign bodies, dead cells, and debris and recruit monocytes/macrophages in response to inflammatory signals. The activation of transcription factors in the long-lived tissue-resident macrophages and/or monocyte-derived macrophages triggers epigenetic modifications, leading to the pathogenesis of chronic diseases. Therefore, the application of an in vitro model using macrophages has been increasingly used to study the antioxidant properties of nutraceuticals. In addition, it makes it possible to take into account the key biological parameters needed to reflect real physiological conditions [8].

## 2. Results

### 2.1. Antioxidant Capacity of Goutweed Extracts Obtained Using Different Solvents

The antioxidant potential of the extracts at 100 mg/L ranged from 6.54% to 15.65%. The highest percentage inhibition of DPPH (2,2-diphenyl-1-picrylhydrazyl) (15.65%) was observed in the extract dissolved in DMSO, which was extracted with water at 90 °C. The lowest inhibition potential was noted with the first extraction method, amounting to 13% for DMSO and 6.6% for water/ethanol. The percentage difference between solvents used in the same extraction method amounted to 41.9%, 40.3%, 56%, and 32.3%, respectively. The results and p-values are presented in Table 1.

The lowest reducing power, determined using the FRAP (ferric-reducing antioxidant power) assay in leaf extracts was found in the first extract for both solvents. In the extract dissolved in water, the result was 177.8 µM Fe(II)/L, whereas for DMSO, it was 152 µM Fe(II)/L. Percentage-wise, the difference amounted to 14.5% and was not statistically significant (*p* > 0.05) (Table 1). The greatest amount of Fe(II) ions was observed in samples extracted with ethanol at 80 °C (water: 416.4 µM Fe(II)/L; DMSO: 302.9 µM Fe(II)/L). Percentage-wise, the difference amounted 27.3% and was not statistically significant, either (*p* > 0.05). In the second extract, the difference amounted to 21.6%, and in the third, it amounted to 12%. The remaining p-values are presented in Table 1.

The highest concentration of polyphenolic compounds in leaf extracts was noted in the fourth extract for both solvents. For the extract dissolved in ethanol, the content was 17.8 mg/L, and for DMSO, it was 14.0 mg/L. Percentage-wise, the difference amounted to 21.5% and was statistically significant (*p* ≤ 0.05) (Table 1). The lowest concentration of phenolic compounds was observed in the first extract, which was made with water at room temperature (water: 5.7 mg/L, DMSO: 5 mg/L). Percentage-wise, the difference amounted to 12.0% and was statistically significant, too (*p* ≤ 0.05). In the second extract, the difference amounted to 14.1%, and in the fluoride third, it was as little as 1%. The total polyphenols content (TPC) based on the dry weight of the extract was 17.79 g/100 g for the 80 °C ethanol extract, which was dissolved in ethanol (17.79 mg/L). Thus, the percentage of polyphenols in the extract is a maximum of 17.79%. The results and p-values are presented in Table 1.

### 2.2. Chemical Composition of the Leaf Extracts (Polyacetylene and Essential Oil)

Goutweed leaf extracts chosen for the in vitro analysis were also tested for the content of polyacetylenes: falcarinol and falcarindiol. Significantly higher levels of both compounds were found in the extracts made at 80 °C, and falcarinol was absent in extracts made at lower temperatures (*p* ≤ 0.05) (Table 2).

In order to characterise the volatile compounds present in freeze-dried leaves, an essential oil was isolated, and its chemical composition was determined. With the use of the GC-FID-MS method, a total of 106 compounds, accounting for more than 87% of the whole oil, were identified in the essential oil of the leaves of *Aegopodium podagraria*.

The *A. podagraria* oil was found to contain many different compounds. The main volatiles represented diverse chemical groups, both oxygenated derivatives of sesquiterpenes (β-caryophyllene epoxide (3.6%), humulene epoxide II (3.5%), khusinol (2.0%), ledene oxide II (1.8%), salvial-4(14)-en-1-one (1.8%)) and monoterpenes (β-pinene (3%), *m*-cymene (1.4%)) as well as oxygenated derivatives of monoterpenes such as linalool (1.8%) and hydrogenated sesquiterpenes (β-bisabolene (1.8%), β-elemene (1.6%)). The essential oil also contained many oxygen derivatives of chain alkanes, predominantly: octanal (4.1%) and heptadecan-2-one (2.4%). Several fatty acids were detected in *A. podagraria*, the main one being palmitic acid (2.3%) (Table 3).

### 2.3. Cell Culture Experiments

#### 2.3.1. Cellular SOD and CAT Activity

Based on the analysis of the composition and antioxidant potential of the extracts, two extracts with the highest potential were selected for cell culture. Catalase (CAT) activity ranged from 41.6 to 491.0 nmol/min/mg, and that of superoxide dismutase (SOD) ranged from 8.7 to 44.7 U/mL. It was observed that the addition of a medium, i.e., DMSO, to cell culture increased enzyme activity, while the addition of fluoride caused its significant inhibition (FDR ≤ 0.05). The addition of leaf extracts was associated with a significant increase in antioxidant enzyme activity compared to the negative control. When fluoride was added to the extracts, enzyme activity was reduced compared to the extracts alone, but it was still significantly higher than in the case of the culture subjected to the oxidative stress-inducing factor (*p* ≤ 0.05).

#### 2.3.2. Cellular Antioxidant Capacity

A similar effect was observed with respect to antioxidant capacity measured by the ABTS (2,2’-azino-bis(3-ethylbenzothiazoline-6-sulfonic acid) assay. The lowest capacity was noted in the culture with sodium fluoride (13.8 mM). The addition of plant extracts, even with the oxidative stress-inducing factor, significantly increased the antioxidant potential of the tested cells (Table 4).

#### 2.3.3. Anti-Apoptotic Activity of Leaf Extracts

**Figure 2:**
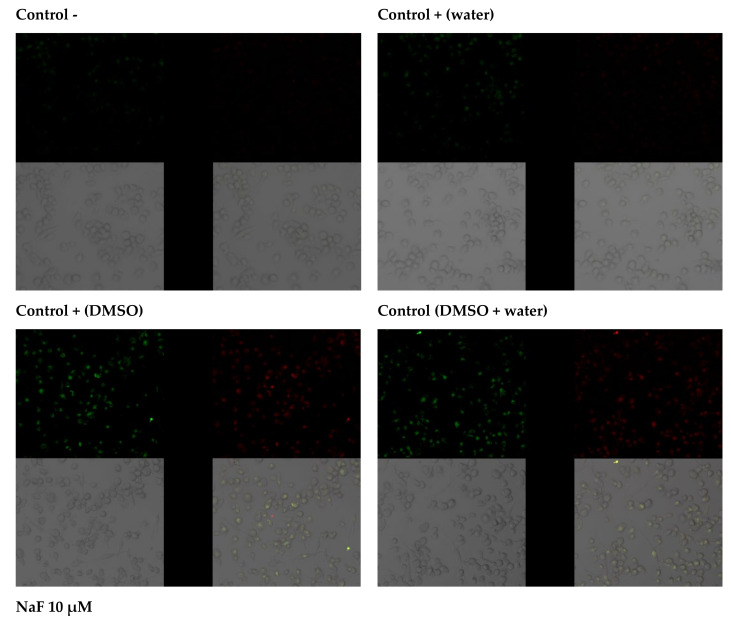

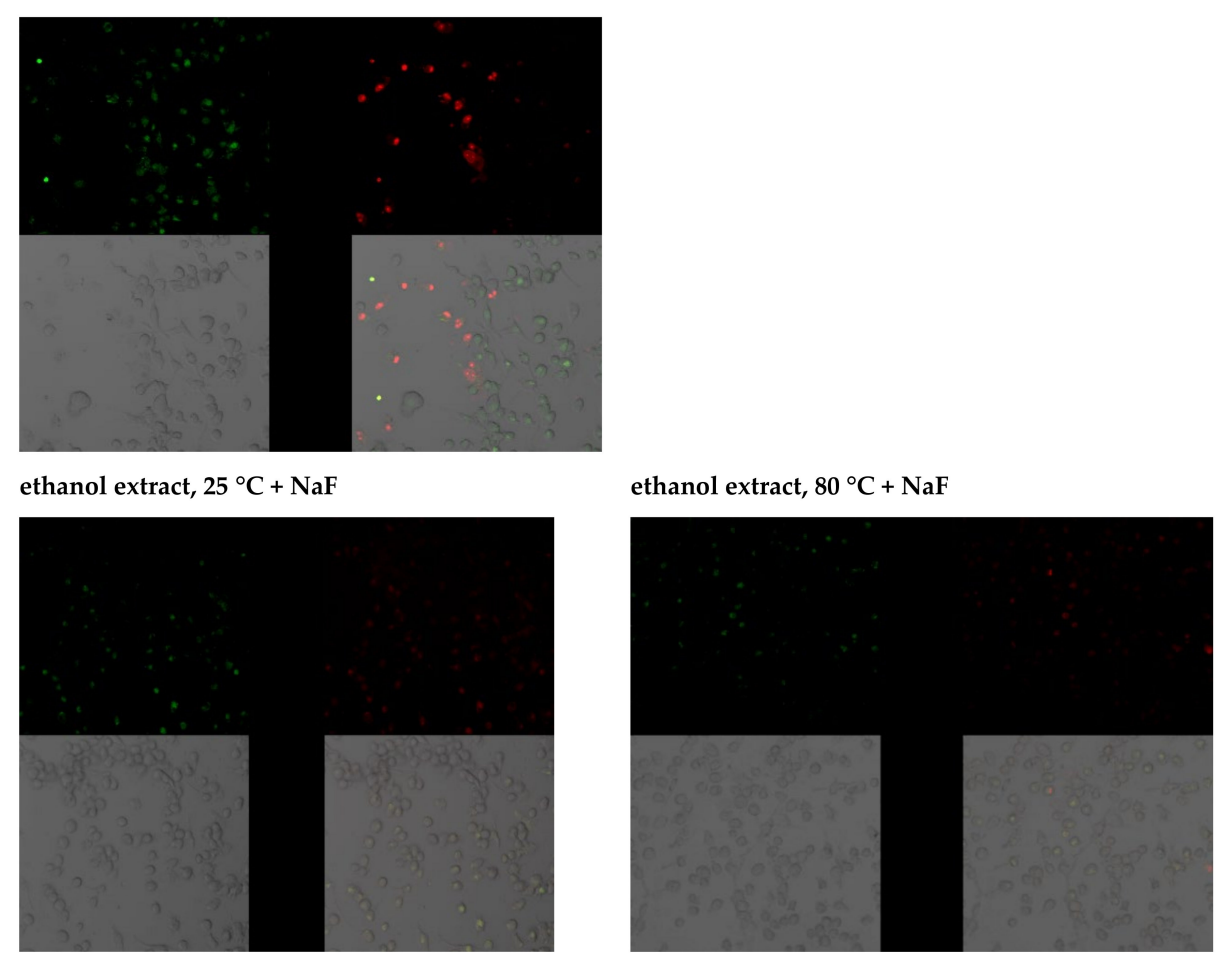

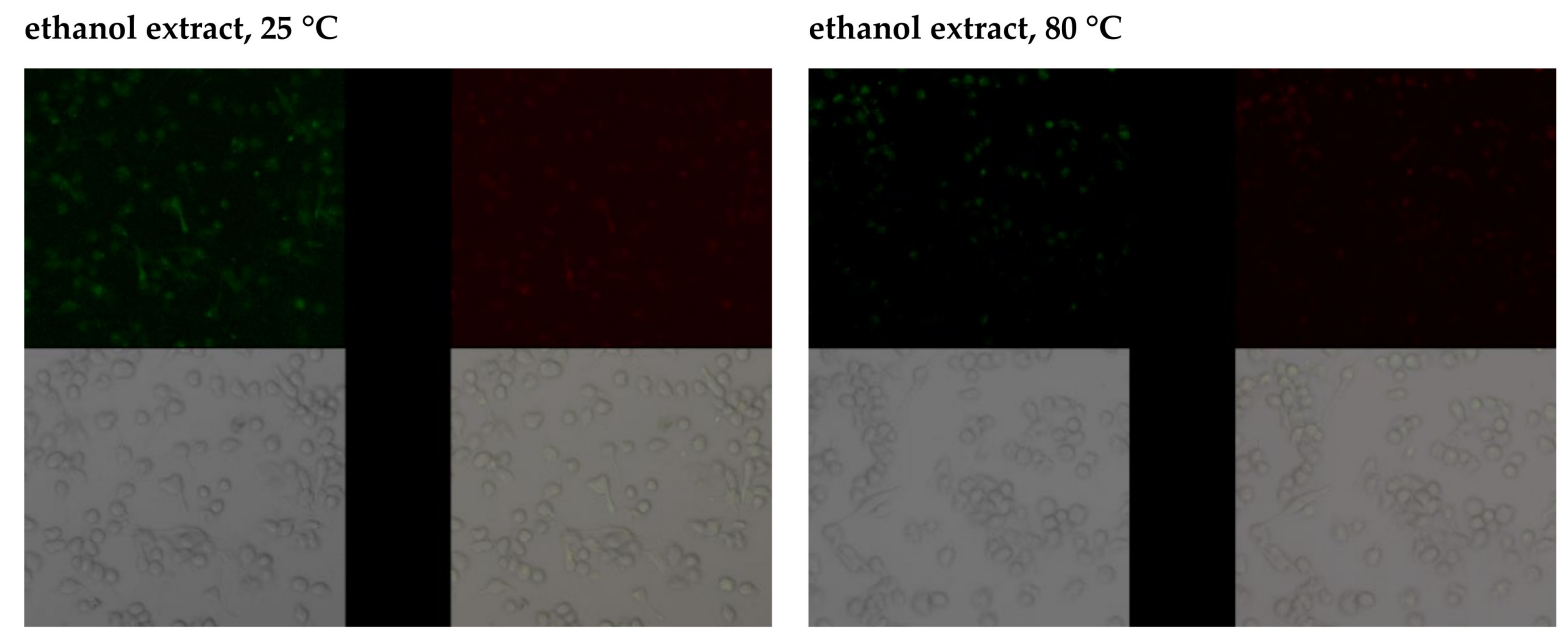
Imaging of apoptosis by confocal microscopy in macrophages cultured with NaF solutions alone or with ethanol extract. THP−1 were cultured with NaF and plant extract for 48 hr as described in Materials and Methods. Cells that are viable are Annexin V-FITC and PI negative; cells that are in early apoptosis are Annexin V-FITC positive and PI negative (green fluorescence); and cells that are in late apoptosis or already dead (necrosis) are both Annexin V-FITC and PI positive (red fluorescence). A dual-pass FITC/rhodamine filter set was applied.

The effects of selected extracts on THP-1 cell survival were determined using the Annexin V-FITC Apoptosis Kit. Data analysis suggests that THP-1 cells grown in an environment containing fluoride at 10 µM die mainly by late apoptosis and necrosis (A+/PI+). The majority of macrophages exposed to DMSO (extract medium) enter the pathway of late apoptosis and necrosis (A+/PI+). However, the observed fluorescence is more intense in cells incubated with sodium fluoride. Cells in the negative control and the control with water as fluoride medium were not observed to enter the apoptotic pathway. A small amount of cells in the early stages of apoptosis (A+/PI−) was noted in THP-1 cells with added extracts; hence, it may be concluded that they did not initiate apoptotic processes. The addition of sodium fluoride to the extracts promoted apoptosis (increased green fluorescence), and cells were shown to undergo late apoptosis and/or necrosis (red fluorescence). In this case, too, cell viability was greater than in the case of cultures with fluoride alone, demonstrating the protective effect of goutweed leaf extracts (Figure 2).

#### 2.3.4. Capacity of Intracellular ROS Decrease

**Figure 3:**
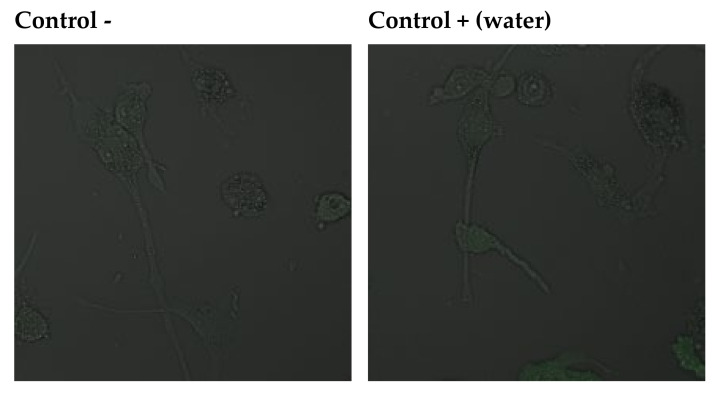

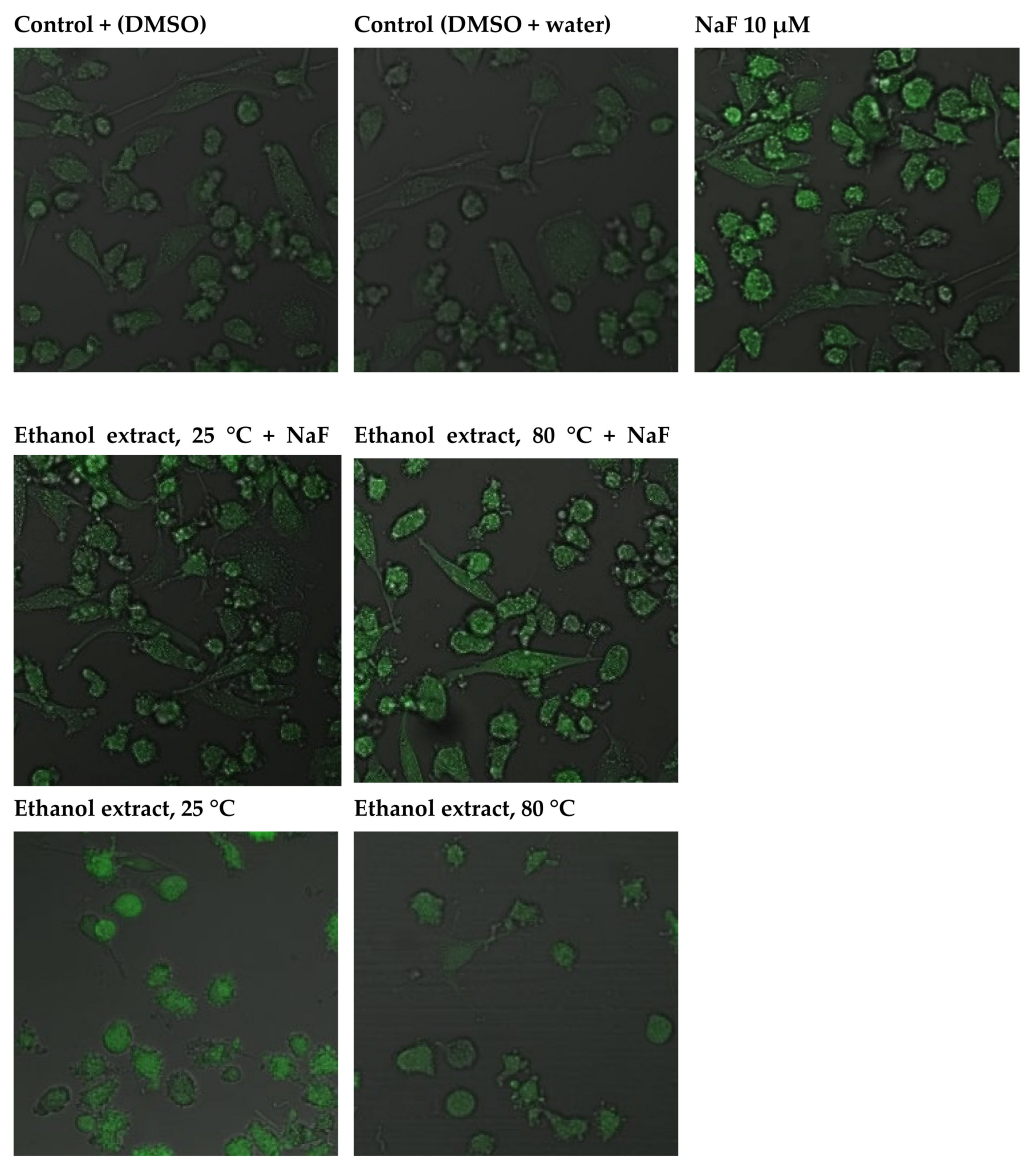
Imaging of cytoplasmatic superoxides detection by fluorescence microscopy in macrophages cultured with NaF solutions alone or with ethanol extract.

The addition of sodium fluoride at 10 µM to the culture resulted in a marked increase in green fluorescence, indicating the enhanced production of free radicals. Increased ROS synthesis was also noted in the control with DMSO and in the control with DMSO and distilled water. A slight increase in the production of free radicals in the cytoplasm was observed after adding plant extracts to the cell culture. Compared to the culture with fluoride or DMSO, the increase in ROS generation was significantly smaller. Introduction into the cell culture of extracts with fluoride led to an increase in the production of free radicals, but it was significantly smaller compared to adding the factor inducing free radical reactions on its own; hence, the extracts were observed to produce an antioxidant effect (Figure 3).

**Figure 4:**
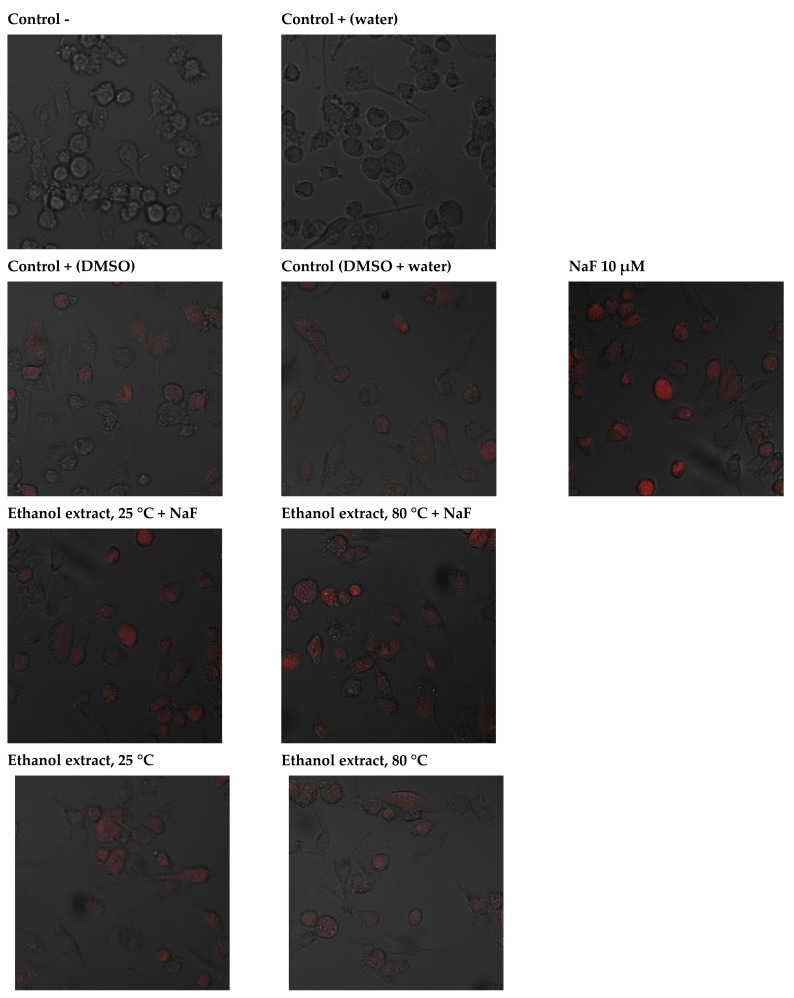
Imaging of mitochondrial superoxides detection by fluorescence microscopy in macrophages cultured with NaF solutions alone or with ethanol extract. THP^−1^ were cultured with NaF and extract for 48 h as described in the Materials and Methods. Detection of mitochondrial superoxide synthesis in macrophages was performed using MitoSOX Red indicator (incubation 10 min/37 °C). The reagent is oxidised only by superoxide, and the oxidation product becomes highly fluorescent upon binding to nucleic acids (red fluorescence).

Fluoride at 10 µM was associated with a dramatic increase in the production of free radicals in the mitochondrion. Increased ROS synthesis was also noted in the control with DMSO and in the control with DMSO and distilled water. The addition of extracts to the cell culture led to a small increase in free radicals in the mitochondrion. Introduction into the cell culture of extracts with fluoride led to an increased production of free radicals, but again, it was lower than in the case of adding fluoride alone, pointing to the protective effect of plant extracts (Figure 4).

## 3. Discussion

Plants are a valuable source of bioactive substances with antioxidant properties. Certain parameters, such as the degree of fragmentation, preservation method (e.g., lyophilisation), extraction conditions (e.g., duration, temperature, mixing) and choice of solvent have a significant impact on these properties [9,10,11]. It is important to learn how these factors affect the antioxidant potential of plant preparations in order to optimise extraction parameters and enhance the antioxidant activity of plant extracts.

### 3.1. Characteristics of the Source Material: Analysis of Essential Oils, Total Polyphenols and Polyacetylenes, and Antioxidant Capacity of Goutweed Extracts Obtained Using Different Solvents

Leaves are vegetative plant parts whose functions include transport, nutrition, as well as conditioning growth. The antioxidant properties of extracts may be attributed to the content of polyphenolic compounds, including flavonoids and phenolic acids, e.g., hydroxycinnamic and chlorogenic acid, essential oils, chiefly mono- and sesquiterpenes, carotenoids and antioxidant vitamins (vitamin E and C), as well as falcarinol and falcarindiol [2,3,4,5,6,7,8,9,10,11,12]. An increased intake of bioactive compounds, including polyphenols, has a positive effect on the body, notably by quenching oxidative stress [13,14]. Hydroxycinnamic acids, such as ferulic, caffeic, and p-coumaric acid, interrupt free radical chain reactions; they also donate electrons or hydrogen and stabilise the emerging phenoxyl radicals [9]. These compounds chelate transition metals, such as copper and iron, which play an essential role in the protection against oxidative stress, and they also inhibit the activity of enzymes producing reactive oxygen species. Chlorogenic acid, found in goutweed, has antioxidant properties, too [9,10,11,12,13,14,15]. Flavonoids, including quercetin and rutin, protect vitamin C against oxidation and take part in the chelation of metal ions, e.g., copper, acting as free radical scavengers as well (primary antioxidants). Individual polyphenolic compounds have different activities that may affect the antioxidant properties of plant extracts [2].

In this study, the extraction method was shown to impact on the overall content of polyphenols and polyacetylenes. TPC ranged from 4.98 to 13.96 mg/L of gallic acid. Higher concentrations were noted in extracts made with ethanol at 80 °C. A similar relationship was observed for polyacetylenes, falcarinol and falcarindiol. Falcarinol content in extracts obtained with water at room temperature was below detection level, while at 80 °C, it amounted to 46.91 ± 3.47. Falcarindiol content ranged from 185.81 ± 19.21 to 474.51 ± 75.74 mg/100g of the extract. Significantly higher levels (*p* ≤ 0.05) were observed in ethanol extracts made at higher temperatures, so it may be concluded that this extraction method improves the composition and properties of the product. Polyacetylenes found in goutweed have powerful anti-inflammatory effects, showing inhibitory activity against cyclooxygenases [5,6,7]. The falcarindiol content in essential oils isolated from leaves amounted to 0.6%, and from stems, it amounted to 0.2% [5]. The effects of falcarinol and falcarindiol on the stress responses were studied in primary myotube cultures. Biphasic responses on cellular stress responses in myotube cultures were investigated by exposing them to various concentrations of falcarinol and falcarindiol for 24 h. At low concentrations (1.6 to 25 μM), polyacetylenes caused a slightly accelerated intracellular ROS formation and increased cGPx transcription. The increased cGPx transcription may be interpreted as an adaptive response to the increased ROS formation. However, ROS formation, was substantially decreased after pre-incubation with both polyacetylenes at 50 and 100 μM, and cGPx transcription was reduced. In conclusion, pre-incubation with low concentrations of both polyacetylenes prior to H_2_O_2_ exposure induced a cytoprotective effect, whereas higher concentrations had adverse effects. Therefore, the effect is dose-dependent [6]. While falcarinol and falcarindiol are the main biologically active compounds in the *Apiaceae* family, research evidence in this scope is still insufficient.

Similar results were observed in clover extracts at 100 mg/L. The TPC for white clover extracts amounted to 14.29 mg/L, and for red clover extracts, it amounted to 19.20 mg/L of gallic acid [16]. In fresh and frozen goutweed leaves, TPC was determined to amount to 700–800 mg 100 g^−1^, with higher content found in frozen product. In turn, the main polyphenols were vanillic acid 3.56 ± 0.15 mg 100 g^−1^ and catechin hydrate 1.96 ± 0.08 mg 100 g^−1^ [17]. It was noted that the total content of phenols and flavonoids was directly proportional to extract concentration. In 10% extracts of goutweed, the total phenolic content was in the range 59.96–134.03 mg/g (of gallic acid equivalent), and it is these compounds that may be largely credited with the antioxidant activity of *Apiaceae* plant extracts. Extracts of *Aegopodium podagraria* L. were found to have the highest total phenolic content compared to other members of the *Apiaceae* family, that is *Centella asiatica* L. and *Meum athamanticum* [18].

Essential oils found in the plants from the *Apiaceae* family, especially monoterpenes, have high antioxidant capacity, which was confirmed in numerous studies [19,20,21,22]. Their properties are associated with reduced oxidative stress due to scavenging free radicals.

A total of 106 compounds, accounting for more than 87% of the whole oil, were identified in the essential oil of freeze-dried leaves of *Aegopodium podagraria* by GC-FID-MS. The dominant compound was spathulenol (15.8%), an oxygenated derivative of sesquiterpene, followed by *n*-octanal (4.1%), β-caryophylleneepoxide (3.6%), humuleneepoxide II (3.5%), and β-pinene (3%). Spathulenol is found, for example, in *Origanum vulgare* and *Psidium guineense*. It has been shown to possess antioxidant, anti-inflammatory, antiproliferative, and antimycobacterial activities [23]. The essential oil also contained many volatiles from different chemical groups. These results are partially consistent with those of other authors, leading to the conclusion that the country of origin and processing method significantly affect the chemical composition of the plant.

Goutweed from Russia collected during flowering contained a significant amount of sabinene (63%) and other essential oil compounds i.e., α-pinene (3.6%), *β*-pinene (3.79%), myrcene (2.17%), α-thujone (0.63%), dehydro-p-cymene (3.39%), and *β*-phellandrene (0.65%) [24]. Most of these compounds belonged to the monoterpene group. In a plant from Siberia (Russia), the essential oil contained α-pinene (13.3%), followed by limonene (9.4%), p-cymene (8.8%), (*Z*)-*β*-ocymene (5.2%), β-pinene (5.0%), germacrene D (4.7%), spathulenol (4.4%), α-thujone (4.2%), perillaldehyde (4.1%), (*E*)-β-caryophyllene (3.9%), (*E*)-caryophylleneoxide (3.4%), and myrcene (3.4%). The concentration of sabinene was only 1.8% [12]. Differences in the composition of essential oils between the leaves and stems of goutweed were observed in Estonia [3]. Higher levels of these bioactive compounds were present in stems (3.8 mg/g) than in leaves (1.7 mg/g). In leaves, the main compounds were monoterpenes, including *β*-pinene (29.4%), limonene (18.4%), α-pinene (15.7%), and γ-terpinene (15.5%). Stems contained mainly monoterpenes (44%), dominated by *β*-pinene (11.1%), limonene (8.2%), *γ*-terpinene (8.2%), and α-pinene (6.6%), as well as sesquiterpenes (29.8%), including germacrene D (15.7%) and (*E*)-α-bergamotene (4.8%) [5]. Our results show that the composition of essential oil from plants growing in Poland is dominated by oxygenated derivatives of sesquiterpenes, while the essential oils from goutweed collected in Russia and Estionia are mainly composed of monoterpenes such as *α*-pinene, *β*-pinene, and sabinene.

The antioxidant potential of goutweed leaf extracts was determined using two spectrophotometric methods: DPPH and FRAP assays. By using four extraction techniques, we were able to identify the most effective extraction method and the best solvent. In this case, too, alcoholic extraction at a higher temperature was associated with higher antioxidant potential. With the DPPH assay, ethanol extraction at room temperature was found to be effective, too. Antioxidant activity, expressed as percentage inhibition of DPPH radical, ranged from 6.54 to 15.11%, and in terms of reducing power, it ranged from 151.98 to 416.42 µM Fe(II)/L. These results confirm that it is best to use several analytical methods for plant extracts. One method, often with limitations, will not fully reveal the potential of the plant. Most scientific studies support the use of higher temperatures to optimise the extraction of plant materials. In the case of our research, higher results for the temperature of 90/80 ℃ were obtained in the FRAP method than in DPPH, but extracts prepared at a higher temperature had a higher concentration of TPC or polyacetylenes, which may also affect the obtained results. These findings are consistent with those made by Valyova et al., who observed the highest antioxidant capacity (DPPH and ABTS) in ethanol extracts, and high antioxidant potential in the plant itself (respectively, EC_50_ 66 μg mL^−1^ for DPPH and IC_50_ 25 μg mL^−1^ for ABTS assay) [25]. However, the authors did not provide information on the part of the plant the extracts were made from, and the concentration used was ten times higher than that used in this study. Similar findings were obtained in a study of extracts (100 μL/mL) made from dry plants by ultrasonic bath extraction method, where IC_50_ amounted to 64.74 0.22 L/mL [26]. The 10% extract of the common goutweed showed very high antioxidant potential at 80% inhibition of DPPH radical, while the 1% extract showed antioxidant potential at ≈15% inhibition of DPPH radical, with EC_50_ amounting to 3.8% [18]. These results are consistent with the present findings. The high antioxidant potential (>90% inhibition of DPPH) of various parts of the common goutweed was also demonstrated by Wróblewska et al., who investigated a range of different extraction parameters. The authors found ultrasonic-assisted extraction with the use of ethanol to be the most advantageous [10]. Additionally, it was confirmed that spathulenol, the main essential oil compound isolated in our study, exhibited the highest antioxidant activities in the DPPH and MDA system compared with the reference standard (IC_50_ values ranging from 26.13 to 85.60 μg/mL) [23].

The duration and temperature of extraction are important parameters for process optimisation, even in order to minimise the cost of energy used in the process. Lyophilisation (freeze drying) was shown not to alter the composition and antioxidant properties of extracts, making it a good method for preserving plant products [27,28]. Moreover, fragmentation of plant material may help shorten extraction time and increase the amount of bioactive compounds released into the extract [28]. Additionally, fragmentation increases the specific surface area, allowing for more effective mixing of the product with the solvent. With regard to the above, the harvested leaves were frozen, freeze-dried, and homogenised.

In the majority of cases, a higher extraction temperature was associated with greater antioxidant activity. The higher antioxidant potential noted in extracts made at 80/90 °C may be related to the increased substance solubility and diffusion coefficient at higher temperatures [28]. The medicinal properties of plant preparations are significantly dependent on the choice of solvent at every stage of research, whether it is used in the extraction process or to dissolve the solution for the purposes of analysis or to obtain the final concentration. What is more, antioxidants may be isolated from plant material using a range of different techniques and solvents because of the chemical diversity of those compounds [29]. In folk medicine as well as in modern phytotherapy, the most common solvents are water and alcohol. They are used to obtain infusions and macerates (aqueous or alcoholic). In cell line studies, DMSO is used among others as a medium introducing the extract into the cells; hence, in our spectrophotometric analyses, different solvents were used (DMSO, water, ethanol) in order to observe the differences arising from the use of different solvents commonly used in phytotherapy and cell models. It was determined that the best extraction method involved the use of ethanol at 80 °C. Extraction with ethanol at room temperature was found to be effective, too. Therefore, it can be concluded that ethanol as an extracting agent increased the antioxidant capacity of extracts to a greater extent than water. Other authors also reported that ethanol, because of its properties, was a better solvent in the case of plant products than water [27]. The observed results may be related to the hydrophilic properties and polarity of bioactive compounds isolated from plants [30]. The varied polarity of extracted components and solvents used may determine the properties of the extract [31]. Ethanol is a moderately polar organic solvent, whereas water is highly polar. In turn, DMSO is a highly polar aprotic solvent. Water is good at dissolving ions and polar molecules but poor at dissolving nonpolar molecules, e.g., organic compounds such as hydrocarbons and their derivatives. Therefore, the chemical composition of extracts determines their properties. The present findings are consistent with those made by Valyova, who studied goutweed extracts made with the use of different solvents, observing the highest antioxidant capacity in ethanol extracts [25]. With respect to FRAP and Folin–Ciocalteau assays, the differences between solvents (DMSO, water/ethanol) were minor and for the most part did not have a significant effect on the antioxidant potential of extracts. In the above analyses, extracts dissolved in water/ethanol had more powerful antioxidant properties than those dissolved in DMSO. The opposite was observed for the DPPH method. DMSO increased the potential in extracts, and the differences between solvents were considerable and statistically significant (*p* ≤ 0.05).

The presented data confirm that goutweed extracts may provide a valuable source of natural antioxidants, including but not limited to polyphenols, essential oils, and polyacetylenes. In addition, determining the effects of various parameters on the health-promoting properties of extracts made it possible to choose the extraction method and temperature maximising their antioxidant potential.

### 3.2. Study of the Antioxidant Response of Macrophages Exposed to Goutweed Extracts

Our study is the first to report on the antioxidant effects of *Aegopodium podagraria* extracts on cell lines. In order to study the cellular antioxidant response of macrophages exposed to goutweed leaf extracts, we determined the total antioxidant capacity by ABTS method, activity of antioxidant enzymes (CAT, SOD), as well as mitochondrial and cytoplasmic generation of free radicals.

Sodium fluoride was used in this study to induce oxidative stress in macrophages. Fluoride toxicity has been well documented, and its mechanism is related, among others, to the disturbance of redox balance. Our study demonstrated that fluoride significantly reduced the total antioxidant capacity measured by ABTS and the activity of antioxidant enzymes: catalase and superoxide dismutase. Additionally, it enhanced oxidative stress by upregulating the production of oxygen free radicals, especially superoxide anion radical in the mitochondrion and cytosol, which has also been observed in other studies [16,17,18,19,20,21,22,23,24,25,26,27,28,29,30,31,32]. It may be concluded that high concentrations of fluoride (10 µM) inhibit antioxidant enzymes and undermine the functionality of the “antioxidant apparatus”. These results are consistent with the reports of other authors, who likewise demonstrated the inhibition of the above enzymes by fluoride [33,34].

Extracts added to cell cultures were shown to effectively quench oxidative stress by boosting the antioxidant capacity of cells (ABTS) as well as the activity of antioxidant enzymes. The addition of extracts into the cell culture unblocked enzymes, which were previously inhibited by fluoride. The elements found in extracts supplied the necessary cofactors for antioxidant enzymes, which were previously blocked by the highly reactive sodium fluoride, facilitating the cell defence system. Polyphenols, polyacetylenes, and essential oils found in extracts may enhance the activity of antioxidant enzymes, including superoxide dismutase, catalase, and glutathione peroxidase, as well as contribute to increasing the concentrations of small-molecule antioxidants, improving the total antioxidant capacity of cells [16]. Similar findings were made in a macrophage study, where sodium fluoride also suppressed antioxidant capacity and inhibited enzyme activity, while extracts of red and white clover effectively unblocked the activity of those enzymes and produced antioxidant effects [16].

In this study, we also determined the mitochondrial and cytoplasmic generation of oxygen free radicals. The increased generation of free radicals, both in the mitochondrion and cytoplasm, was observed in the presence of DMSO and sodium fluoride; however, extracts were shown to exert a protective effect against the free radical activity of sodium fluoride. Research findings are conclusive that sodium fluoride at 10 µM initiates cell death. The proapoptotic and necrotic effects of fluoride are related to initiating oxidative stress and boosting the production of superoxide anion radicals. Fluoride also alters the activity of antioxidant enzymes and initiates inflammation, interfering with the defensive mechanisms in cells [33,34,35]. Apoptosis and necrosis initiation was also observed in THP-1 macrophages with DMSO (1%) added to cell culture as a goutweed extract medium. Our study is consistent with the literature, confirming the negative effect of this solvent even in small concentrations [36,37,38,39]. Therefore, DMSO is not a neutral solvent for cells, and its use—even in small concentrations (>0.5%)—as a medium for plant extracts interferes with their action. The effects of low concentrations of DMSO (0.1–2%) on the antioxidant status in plant cells were investigated in an in vitro study. Elevated stress levels were observed in direct proportion to the concentration of DMSO added to the cell culture [40]. In this study, the addition of DMSO as a control for extracts in macrophages enhanced the antioxidant capacity of cells, as well as catalase activity, while at the same reducing the activity of superoxide dismutase compared to the negative control. This solvent initiated the generation of reactive oxygen species, at the same inducing oxidative stress, which increases the activity of catalase—a free radical scavenging enzyme. Therefore, it may be concluded that DMSO, even in a low concentration (1%), has an adverse effect on the redox balance and enzyme activity.

## 4. Materials and Methods

### 4.1. Plant Material

**Figure 5:**
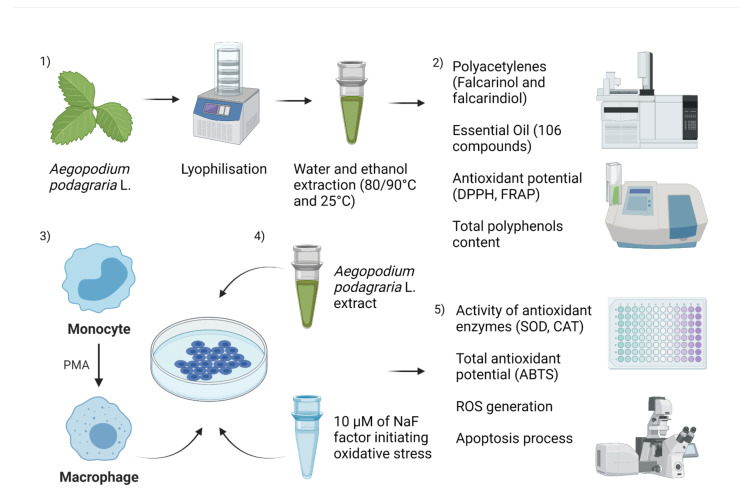
Methodology used to study *Aegopodium podagraria* L. extracts. Created with BioRender.com.

Leaves of goutweed (*Aegopodium podagraria* L.) gathered before flowering in June from green areas (53°28′11.002″ N, 14°29′46.7982″ E) served as the study material for this research. The harvested plant (2 kg) was assessed by a botanist. The collected leaves underwent lyophilisation in a lyophilisator (0.735 mmHg/−20 °C; Alpha 1-2 LD plus) and were then subjected to homogenisation by grinding to a powder in a food homogeniser (FOSS 2094). The steps of the study are presented in Figure 5.

### 4.2. Preparation of an Aqueous Extract

Distilled water (150 mL) at 90 °C or 25 °C was poured over approximately 1.5 g of dried goutweed powder. A closed conical flask with the mixture was placed on a vortex mixer and gently shaken (180 RPM) for 10 min. The mixture was cooled down to room temperature and filtered. From the obtained filtrate, water was evaporated under reduced pressure. The obtained aqueous extract was placed in a plastic vial and then stored at −20 °C until used [16].

### 4.3. Preparation of Ethanol Extract

Approximately 1.5 g of dried goutweed powder was transferred into 150 mL of 96% ethanol (Chempur, Poland). The mixture was kept for 30 min at boiling point (80 °C) or at 25 °C in a water bath under a condenser. In order to maintain a uniform boiling process, a few boiling stones were added to the flask. Then, the mixture was cooled down to room temperature and filtered. Alcohol was subsequently evaporated under reduced pressure. The obtained alcohol extract was placed in a plastic vial and then stored at −20 °C until use [16].

### 4.4. Preparation of Extracts for Analysis

Extracts were dissolved in 96% ethanol (Chempur, Poland) (for ethanol extracts), in distilled water (for aqueous extracts) and in DMSO (Sigma-Aldrich, Poland) to compare solvents used in phytotherapy with a cellular carrier (DMSO) at the 100 mg/l concentration [16,17,18,19,20,21,22,23,24,25,26,27,28,29,30,31,32].

### 4.5. Antioxidant Activity of Extracts by the DPPH Method

The antioxidant activity of samples was measured spectrophotometrically using synthetic radical DPPH (2,2-diphenyl-1-picrylhydrazyl) (Sigma-Aldrich, Poznań, Poland) and an Agilent 8453UV spectrophotometer. First, 96% ethanol, 1 mL of 0.3 mM solution of DPPH in 96% ethanol, and 0.1 mL of the test sample were introduced into the vial with a v/v ratio of 29:10:1. After mixing, the solution was placed for 30 min in a dark place. During this time, the so-called A0 solution was prepared by mixing 96% ethanol and 0.3 mM solution of DPPH in a v/v ratio of 3:1. As a reference solution, 96% ethanol was used. Before measurement, the contents of the vial were thoroughly mixed, poured into quartz cuvettes, and spectral absorbance was immediately measured at 518 nm [41]. All assays were performed in triplicate.

### 4.6. Determination of the Reduction Potential of Extracts by the FRAP Method

The FRAP method, used to determine the total reduction potential, tests the capability of the test sample to reduce Fe^3+^ ions to Fe^2+^ ions. These ions are complexed by TPTZ (2,4,6-tris(2-pyridyl)-1,3,5-triazine) (Sigma-Aldrich, Poznań, Poland), creating an intense blue colour with the maximum absorbance at 593 nm. The antioxidant capacity of the sample is determined by comparing changes in absorbance ΔA with the value ΔA of the standard solution Fe^2+^ (calibration curve). The FRAP unit represents the ability to reduce 1 mole Fe^3+^ to Fe^2+^ [42,43,44]. The solution was mixed and incubated for 4–5 min in a laboratory drier at 37 °C. Then, the solution was mixed thoroughly and transferred to a cuvette for absorbance at 593 nm. All assays were performed in triplicate.

### 4.7. Determination of Polyacetylenes

Falcarinol and falcarindiol were determined by gas chromatography coupled with mass spectrometry (GC-MS QP2010 PLUS, Shimadzu, Kyoto, Japan). Ethanol extracted samples were dried and amounts of ca. 50 mg were dissolved in ethanol. Solutions were centrifuged and analysed chromatographically. Compounds were separated using a ZB-5MSi (30 m × 0.25 mm × 0.25 μm) capillary column (Phenomenex Inc., Torrance, CA, USA) with helium as a carrier gas (0.9 mL/min). The injector temperature was set at 230 °C, and the column temperature was programmed as follows: 70 °C, a subsequent increase to 230 °C at the rate of 15 °C/min., to 310 °C at the rate of 3 °C/min., and then held for 10 min. The GC-MS interface and ion source temperatures were set at 240 °C, ionisation energy was set at 70 eV. The total ion current (TIC) mode (40–200 m/z range) was used. Compounds were identified on the basis of their mass spectra compared with mass spectral libraries (NIST08 library, Shimadzu, Kyoto, Japan). A quantitative analysis was carried out in SIM mode (129 m/z) with a calibration curve using falcarinol (Sigma-Aldrich, Poland) as an external standard.

### 4.8. Determination of the Total Phenolic Content (TPC) in Extracts

Polyphenol content was determined using the Folin–Ciocalteu reagent. First, 5.0 mL of a 10% Folin–Ciocalteu (Chempur, Poland) solution and 1.0 mL of the test sample were successively introduced into a vial. The sample was shaken vigorously, and after 5 min, 4.0 mL of 7.5% Na2CO3 (Chempur, Poland) solution was added. The prepared solution was incubated for 60 min at room temperature. Absorbance at 765 nm was measured. The polyphenol content was determined from the calibration curve using gallic acid (Sigma-Aldrich, Poznań, Poland) as a reference standard. All tests were performed in triplicate. The results are shown in mg/L gallic acid (GAE) [11].

### 4.9. Isolation of Essential Oil

Essential oil from the aerial parts (freeze-dried leaf) of *Aegopodium podagraria* L. was obtained by hydrodistillation using a Clevenger-type apparatus. Hydrodistillation was conducted for 4 h using 150 g of dried plant material. The yellowish essential oil was dried over anhydrous magnesium sulphate and stored at 4 °C in the dark until tested and analysed.

### 4.10. Identification of Essential Oil Constituents

Constituents of the essential oil were identified based on their mass spectra compared with those from mass spectra libraries: NIST 2012, Wiley Registry of Mass Spectral Data 8th edition, and MassFinder 4.1, along with the relative retention indices (RI) on DB-1 column (available from MassFinder 4.1).

### 4.11. Analysis of Essential Oil

GC-MS-FID analysis of the essential oil was performed on a Trace GC Ultra Gas Chromatograph coupled with a DSQII mass spectrometer (Thermo Electron, Waltham, MA, USA). Simultaneous GC-FID and GC-MS analyses were performed using a MS-FID splitter (SGE Analytical Science, Ringwood, VIC, Australia). The mass range was 33–550 amu, ion source-heating: 200 °C; ionisation energy: 70 eV. One microlitre of essential oil solution (80% v/v) diluted in pentane/diethyl ether was injected in split mode at a split ratios (50:1). Operating conditions: capillary column Rtx-1 MS (60 m × 0.25 mm i.d., film thickness 0.25 µm), and temperature program: 50 °C (3 min)—300 °C (30 min) at 4 ◦C/min. Injector and detector temperatures were 280 °C and 300 °C, respectively. The carrier gas was helium (constant pressure: 300 kPa). The relative composition of each essential oil sample was calculated from GC peak areas according to total peak normalisation, which is the most popular method used in essential oil analysis.

### 4.12. Cell Cultures

The monocytic cells, THP-1 line of the American Type Collection (ATCC, Rockville, MD, USA), cultivated on 6-well plates (2 × 10^6^ cells), were differentiated into macrophages by adding 10 µL of phorbol ester (PMA, 100 nM) to the culture medium RPMI (Wytwórnia Szczepionek i Surowic, Lublin, Poland) that included 10% of the foetal bovine serum (FBS) (Gibco, Paisley, UK), penicillin (100 U/mL), and streptomycin (100 mg/mL) (Sigma—Aldrich, Poznań, Poland). The cells were incubated in standard conditions (37 °C and 5% CO_2_) for 24 h. Next, the adherent macrophages were washed three times with PBS. The cells were incubated for 48 h with the factors: NaF at a concentration of 10 μM, causing oxidative stress [16] and/or extracts of goutweed with a final concentration of 100 mg/mL [16,17,18,19,20,21,22,23,24,25,26,27,28,29,30,31,32]. Plant extracts were dissolved in DMSO at a concentration not exceeding 1%. Two extracts with the highest antioxidant potential obtained in spectrophotometric studies were selected for in vitro tests. The positive control for fluorine was distilled water, for the extracts from the plant, it was DMSO, while for both variants introduced to the culture, it was simultaneously water and DMSO. The concentrations of the added fluorides were chosen based on the results of human serum studies carried out by other authors. After 48 h of incubation, the cells were scrapped and centrifuged, and the resulting cell pellets were used in spectrophotometric measurements. The experiment was repeated 5 times. Protein concentration was measured using the Micro BCA Protein Kit (Thermo Fisher Scientific, Waltham, MA, USA).

### 4.13. Measurement of the Total Antioxidant Potential (ABTS) and the Activity of Antioxidant Enzymes

Antioxidant potential was measured using the Antioxidant Assay Kit (Cayman Chemical Company). The kit measures the total antioxidant capacity of cell lysates. The activity of antioxidant enzymes was measured using the Superoxide Dismutase Assay Kit, Catalase Assay Kit (Cayman Chemical Company). The antioxidant potential was measured spectrophotometrically at 405 nm and presented in the form of mM. Catalase activity was measured spectrophotometrically at 540 nm and presented in nmol/min/mL/protein. The activity of superoxide dismutase was measured spectrophotometrically by estimating the amount of the superoxide anion in room temperature at 440 nm and presented in [U/mL]. The intensity of the reaction produced in the reaction was measured in a microplate reader (Biogent).

### 4.14. Imaging of Intracellular ROS Generation

**Figure 6:**
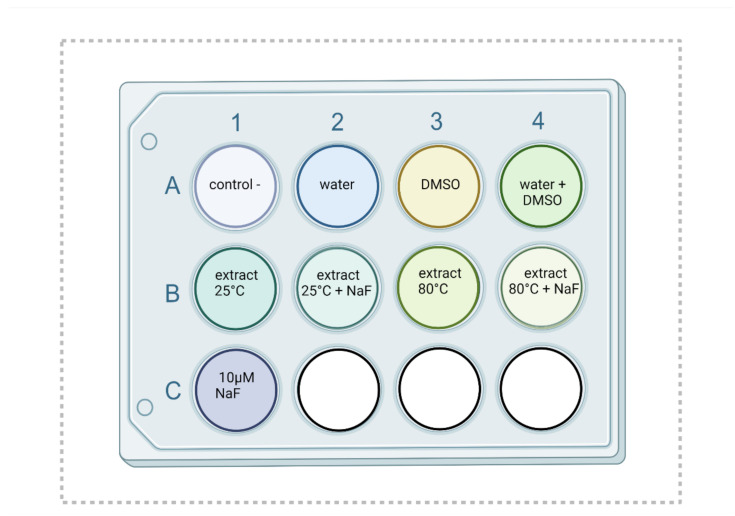
Cell culture with factors incubated with THP-1 line macrophages. The experiment was repeated 5 times.

The intracellular generation of reactive oxygen species (ROS) was visualized by MitoSOX™ Red reagent (Thermo Fisher Scientific, Waltham, MA, USA), which is a novel fluorogenic dye for highly selective superoxide detection in the mitochondria of live cells. Cells were loaded with 5 µM MitoSOX™ reagent working solution and incubated for 10 min at 37 °C (humidified 95% air/CO_2_ atmosphere at 37 °C), protected from light. The concentration of the MitoSOX™ reagent working solution should not exceed 5 μM. After incubation, the cells were washed gently three times with a warm buffer at room temperature, and the preparations were examined under a confocal microscope (Olympus, SV 1000). Once in the mitochondria, MitoSOX™ Red reagent is oxidised by superoxide but not by other reactive oxygen (ROS) or nitrogen (RNS) species, showing red fluorescence. The oxidation product becomes highly fluorescent upon binding to nucleic acids (excitation at 510 nm, emission at 580 nm) (Figure 6).

### 4.15. Imaging of Cytoplasmic ROS Generation

The production of cytoplasmic ROS was visualised using DCFH-DA (Sigma-Aldrich, Poland). DCFH-DA is a nonpolar dye that is converted into the polar derivative DCFH by cellular esterases. DCFH is non-fluorescent but switches to highly fluorescent DCF when oxidised by intracellular ROS or other peroxides (excitation at 485 nm, emission between 500 and 600 nm). DCFH-DA at a concentration of 5 μM was added to the cells. After incubation (30 min at 37 °C), the cells were washed with culture medium at room temperature, and the preparations were examined under a confocal microscope (Olympus, SV 1000, Waltham, MA, USA).

### 4.16. In Vitro Imaging of Apoptosis

Cells (1 × 10^5^ cells/well) were incubated with fluoride solutions alone or with an ethanol or water extract on microscope slides according to the aforementioned procedure. After 48 h of incubation, the cells were rinsed with PBS, suspended in a binding buffer, and stained with 5 ng/mL Annexin V-FITC and 5 ng/mL propidium iodide for 5 min in the dark. Cells that are viable are Annexin V-FITC and PI negative; cells that are in early apoptosis are Annexin V-FITC positive and PI negative (green fluorescence); and cells that are in late apoptosis or already dead (necrosis) are both Annexin V-FITC and PI positive (red fluorescence). A dual-pass FITC/rhodamine filter set was applied. In vitro imaging of apoptosis and necrosis was obtained using confocal microscopy (Olympus, SV 1000, Waltham, MA, USA).

### 4.17. Statistical Analysis

The observations were subjected to a statistical analysis using the Statistica 13.5 software from StatSoft (Poland). In all the experiments, three samples were analyzed, and all the assays were carried out at least in triplicate. The distribution of results was tested with the Shapiro–Wilk test. To evaluate the differences between the studied parameters, the non-parametric Mann–Whitney U-test or the Tukey test was used, depending on the distribution. Correlation analyses were performed using the Pearson test or the Spearman test, depending on the distribution. The statistical significance level was set at *p* ≤ 0.05. To control type I errors, the false discovery rate (FDR) approach was used. The calculations were performed using the p. adjust function of the stats package in R (R Foundation for Statistical Computing, Vienna, Austria, https://cran.r-project.org, accessed on 12 June 2021).

## 5. Conclusions

Common goutweed (*Aegopodium podagraria* L.) has antioxidant properties, which may differ depending on a range of factors. Extraction at 80 ℃ and the use of ethanol as a solvent increased the antioxidant capacity of the extract, which may be related to the presence of polyacetylenes, polyphenols, and essential oils. These properties have been confirmed for the first time in cell lines, demonstrating that the extracts unblock antioxidant enzymes and provide protective effects against sodium fluoride toxicity. DMSO (1%) as a solvent does not have a significant effect on antioxidant capacity determined by FRAP and TFC assay, but when used as a medium in cell cultures, it has proapoptotic activity, disturbing the redox balance. The potential applications of the extracts can be sought in phytotherapy for the support and treatment of conditions involving enhanced oxidative stress, e.g., inflammatory conditions, cardiovascular diseases, neurodegenerative diseases, and even obesity. However, there is a shortage of in-depth research studies presenting the relative content of bioactive compounds in individual parts of the goutweed plant, which shows potential for further research in this scope. In future research, it would be important to consider different, lower extract concentrations and to study other parts of the plant. Another important aspect is the bioavailability and antioxidant properties of the digestive extracts available to the colon, as well as the loss of bioactive compounds during the digestion process. Additionally, it seems necessary to analyse goutweed composition further, including the quantitative and qualitative determination of polyphenolic compounds.

## Figures and Tables

**Table 1:** Antioxidant potential (DPPH, FRAP); total polyphenol content (TPC) in *Aegopodium podagraria* leaf extracts (100 mg/L).

Extraction	Solvent	DPPH			FRAP			TPC		
		[%]			[uM Fe(II)/L]			[mg/L] Gallic Acid (GAE)/[g/100 g DW]		
water 25 °C^z^	water	9.65	±	1.29 *^y,x,v,f,h^	177.79	±	32.31 *^y,x,w^	5.66	±	0.45 *^y,x,w,v,f,h^
water 90 °C^y^	water	6.54	±	0.96 * ^z,x,w,v,u,h,f^	331.03	±	46.45 *^z^	12.85	±	0.22 *^z,w,u^
ethanol 25 °C^x^	ethanol	13.92	±	1.07 *^z,y,w,u,f^	333.55	±	123.55 *^z^	13.73	±	0.38 *^z,u^
ethanol 80 °C^w^	ethanol	10.83	±	1.40 *^y,x,v,f,h^	416.42	±	36.85 *^z,v,u,f,h^	17.79	±	1.02 *^z,y,u,h^
water 25 °C^v^	DMSO	9.71	±	0.36 *^z,y,w,u^	151.98	±	14.50 *^w^	4.98	±	0.46 *^z,u^
water 90 °C^u^	DMSO	15.11	±	0.46 *^y,w,v,f,h^	259.68	±	14.22 *^w^	11.03	±	0.49 *^y,x,w,u,f,h^
ethanol 25 °C^f^	DMSO	14.42	±	2.10 *^z,y,x,w,u^	293.91	±	96.06 *^w^	13.83	±	3.81 *^z,u^
ethanol 80 °C^h^	DMSO	15.65	±	0.55 *^z,y,w,u^	302.86	±	44.66 *^w^	13.96	±	0.23 *^z,w,u^

* FDR *p* ≤ 0.05 between type of extract, *p* ≤ 0.05 between type of extract: z–water 25 °C, solvent–water; y–water 90 °C, solvent–water; x–ethanol 25 °C, solvent–water; w–ethanol 80 °C, solvent–water; v–water 25 °C, solvent–DMSO; u–water 90 °C, solvent–DMSO; f–ethanol 25 °C, solvent–DMSO; h–ethanol 80 °C, solvent–DMSO; DW–dry weight.

**Table 2:** Polyacetylene content in *Aegopodium podagraria* leaf extracts (100 mg/L).

Extract	Falcarinol mg/100g	Falcarindiol mg/100g
ethanol 25 °C ^f^	nd	185.81 ± 19.21 *^h^
ethanol 80 °C ^h^	46.91 ± 3.47 *^f^	474.51 ± 75.74 *^f^

* FDR *p* ≤ 0.05 between type of extract: f–ethanol 25 °C; h–ethanol 80 °C.

**Table 3:** Composition of the essential oil of freeze-dried leaves of *Aegopodium podagraria*.

Compound	Amount [%]	RI exp. *
*n*-Hexanal	0.4	772
*n*-Heptanal	1.1	876
Benzaldehyde + *α*-Pinene	0.4	927
Octane-2,3-dione	0.1	959
6-Methylhept-5-ene-2-one	0.8	961
*β*-Pinene	**3.0**	967
2-Pentylfuran	0.5	976
*n*-Octanal	**4.1**	980
2-Phenylethanal	0.2	1006
*m*-Cymene	**1.4**	1010
Limonene	0.4	1019
(*E*)-Ocimene	0.1	1025
(*E*)-Oct-2-enal	0.2	1031
3-Ethyl-2-methylhexa-1,3-diene	0.1	1043
*γ*-Terpinen	0.4	1048
*cis*-Linalol oxide (furanoid)	0.2	1057
(*E*,*E*)-Octa-3,5-dien-2-one	0.3	1065
Nonan-2-one	1.1	1070
*n*-Heptanoic acid	0.1	1075
(*E*)-6-Methylhepta-3,5-dien-2-one	0.1	1078
*n*-Nonanal	0.8	1082
Linalool	**1.8**	1085
Nopinone	0.4	1106
Non-3-en-2-one	0.1	1116
*trans*-Pinocarveol	0.9	1123
(*E*,*Z*)-Nona-2,6-dienal	0.1	1126
*cis*-Verbenol	0.3	1128
(*E*)-Non-2-en-1-al	0.5	1135
α-Pinocarvone	0.3	1138
*trans*-Verbenol	0.1	1146
*endo* Borneol	0.2	1149
*p*-Cymen-8-ol	0.3	1160
Terpinen-4-ol	0.8	1162
Myrtenal	0.3	1169
*α*-Terpineol	0.4	1172
Myrtenol	0.8	1179
Caprylicacid	1.2	1184
2,6,6-Trimethylcyclohex-1-ene-1-carbaldehyde	0.1	1194
*trans*-Carveol	0.1	1196
Thymol methylether	0.1	1209
*p*-Cumical dehyde	0.1	1211
(*Z*)-Hex-3-enyl isovalerate	0.1	1218
Geraniol	0.2	1236
(*E*)-Dec-2-en-1-al	1.0	1238
*p*-Menth-4(8)-en-9-ol	0.1	1249
Pelargonic acid	0.9	1268
Carvacrol	0.5	1277
Dihydroedulan I	1.3	1282
Dihydroedulan II	1.2	1286
(*E,E*)-Deca-2,4-dienal	0.1	1289
Tridecane	0.7	1300
*α*-Cubebene	0.1	1349
Non-2-enoic acid	0.6	1353
Capric acid	0.5	1365
*α*-Cubebene	0.2	1373
*α*-Ylangene	0.2	1377
*β*-Elemene	**1.6**	1389
(*E*)-Dec-2-enoic acid	0.3	1397
(*E*)-α-Ionone	0.1	1407
(*E*-β-Caryophyllene	0.7	1419
β-copaene	0.3	1429
*trans*-α-Bergamotene	0.4	1434
(*Z*)-β-Farnesene	0.3	1437
(*E*)-β-Farnesene	0.4	1447
α-Himachalene	0.2	1450
α-Humulene	0.4	1453
5,6-Epoxy-β-ionone	0.7	1463
β-Ionone	1.0	1466
α-Curcumene + γ-Muurolene	0.5	1472
Germacrene D	1.0	1479
(3*Z*,*6E*)-*α*-Farnesene	0.3	1482
*α*-Selinene	0.3	1484
*γ*-Muurolene	0.2	1490
2,4-Ditert-butylphenol	1.3	1493
*β*-Bisabolene	**1.8**	1503
*γ*-Cadinene	0.2	1508
*δ*-Cadinene	0.7	1516
*α*-Calacorene	0.4	1531
Isoaromadendreneepoxide	1.1	1545
*cis*-Sesquisabinenhydrate	0.2	1553
1,5-Epoxysalvial-4(14)-ene	0.4	1559
Spathulenol	**15.8**	1576
*β*-Caryophylleneepoxide	**3.6**	1578
Salvial-4(14)-en-1-one	**1.8**	1585
Humuleneepoxide II	**3.5**	1601
Aristoleneepoxide	0.4	1604
Isopropyllaurate	0.3	1614
7-Hydroxyfarnesen	0.8	1616
Widdrol	0.7	1630
*trans*-Longipinocarveol	0.5	1640
α-Cadinol	0.4	1643
Ledeneoxide II	**1.8**	1659
Khusinol	**2.0**	1673
2-Ethylhexyl benzoate	0.4	1768
Myristoleic acid	0.2	1797
diIisobutylphtalate (artifact)	0.1	1827
Heptadecan-2-one	**2.4**	1832
Decan-2-yl benzoate	0.2	1834
Methylpalmitate	0.5	1908
di-Butyl phthalate (artifact)	0.2	1918
Palmitic acid	**2.3**	1960
Phytol	1.1	2101
Tricosane	0.2	2298
Pentacosane	0.1	2499
Sum of identified	**87.0**	

* RI exp–experimental retention index calculated on non-polar column; artifacts originate from plastic packaging.

**Table 4:** Antioxidant potential in THP-1 cell cultures with *Aegopodium podagraria* leaf extracts (ABTS; enzyme activity: SOD—superoxide dismutase, CAT—catalase).

Sample	ABTS			SOD			CAT		
	[mM]			[U/mL]			[nmol/min/mL/Protein]		
Negative control ^a^	14.7	±	0.2 *^b,c,d,f,g,h,i^	31.4	±	6.4 *^c,d,e,h,i^	51.3	±	8.1 *^b,c,d^
Water (NaF solvent) ^b^	36.7	±	7.8 *^a,c,f,g,h,i^	30.1	±	3.1 *^c,d,e,h,i^	138.7	±	5.0 *^a,d,e,f^
DMSO (extract solvent) ^c^	67.0	±	6.6 *^a,b,d,e,f,g,h^	11.4	±	2.4 *^a,b,f,g^	203.5	±	30.2 *^a,e,f,g,h,i^
DMSO + water (extract and NaF solvent) ^d^	49.7	±	5.0 * ^a,c,e,f,g,h,i^	12.3	±	1.2 *^a,b,f,g^	219.4	±	55.5 *^a,b,e,f,g,h,i^
NaF 10 µM ^e^	13.8	±	3.2 *^b,c,d,f,g,h,i^	8.7	±	1.5 *^a,b,f,g^	46.3	±	9.3 *^b,c,d^
ethanol extract 25 °C ^f^	113.0	±	8.2 *^a,b,c,d,e,g,i^	44.7	±	12.4 *^c,d,e,h,i^	491.0	±	12.8 *^a,b,c,d,e,g,h,i^
ethanol extract 25 °C + NaF ^g^	84.4	±	9.5 *^a,b,c,d,e,f,h,i^	37.8	±	10.9 *^,d,e,h,i^	245.2	±	22.0 *^a,b,e,f,h,i^
ethanol extract 80 °C ^h^	99.3	±	3.1 *^a,b,c,d,e,g,i^	10.0	±	2.6 *^f,g^	272.1	±	9.6 *^a,b,e,f,g,i^
ethanol extract 80 °C + NaF ^i^	69.4	±	5.1 *^a,b,d,e,f,g,h^	12.0	±	2.8 *^a,b,f,g^	140.1	±	31.9 *^a,d,e,f^

* FDR *p* ≤ 0.05 between type of extract: a—Negative control, b—Water (NaF solvent), c—Water (NaF solvent), d—DMSO (extract solvent), e—DMSO + water (extract and NaF solvent), f—NaF 10 µM, g—ethanol extract 25 °C, h—ethanol extract 80 °C, i—ethanol extract 80 °C + NaF; *n* = 5.

## Data Availability

Not applicable.
